# A randomized cross-over study comparing the performance of HD integra™ central concentrate system versus pre-produced concentrate in hemodialysis

**DOI:** 10.1186/s12882-017-0537-2

**Published:** 2017-04-03

**Authors:** K. Fauziah, K. W. Go, A. Ghazali, M. Zaki, T. O. Lim

**Affiliations:** 1 Ipoh Specialist Hospital, Ipoh, Malaysia; 2 Kuala Lumpur Hospital, Kuala Lumpur, Malaysia; 3 International Medical University, Kuala Lumpur, Malaysia

**Keywords:** Chronic kidney disease, Dialysis, Dialysis fluid, Dialysate, Concentrate, Central concentrate system

## Abstract

**Background:**

Pre-produced bicarbonate concentrates (PPC) are still widely used in developing countries despite its cost and risk but Central Concentrate System (CCS) is lacking in data to support its wider adoption.

**Methods:**

We conducted an 8-week randomized crossover study on 16 Hemodialysis machines to compare CCS versus PPC. Performance is assessed by solute concentrations while safety is assessed by microbial count, endotoxin level and adverse event reporting.

**Results:**

Microbial counts and endotoxin levels were monitored on 48 occasions during the 8-week study for the CCS arm of the study. The levels were all below the action limit during the study. No patient reported any adverse events. Dialysate Sodium, Chloride and Bicarbonate concentrations were measured on a total of 128 occasions for each arm of the study. The relative deviations of Sodium, Chloride and Bicarbonate concentration were within ±5% of their nominal values for both. The 95% Confidence Intervals for the ratio of the mean solute concentrations on the CCS to PPC lie within the tolerance limit of ±5%.

**Conclusion:**

Modern CCS is bacteriologically safe and its performance statistically equivalent to PPC.

## Background

The dialysis fluid or dialysate required for Hemodialysis (HD) is produced continuously during treatment by mixing purified water, an Acid (AC) and a Bicarbonate concentrates (BC). There are currently 4 choices of concentrate products:

1. Ready-to-use liquid concentrates which are pre-produced in a factory (PPC), packaged in 10-L plastic containers and transported to individual HD facilities. PPC is not only costly but also incurs microbial contamination risk as well as considerable environmental cost and occupational safety risk (see below). Its use has been largely abandoned in developed countries, but is still widely used in developing countries including Malaysia where almost all HD facilities use PPCs which are supplied by 5 domestic manufacturers.
2. Powder concentrate in cartridge. Even though this is costlier, the powder form is free from the risk of microbial contamination. It is widely used in Europe but the cartridge is not universally suitable for all types of hemodialysis machines.
3. Central Delivery System which produces the concentrate (CCS) or dialysate (CDS) on-site within a HD facility. The solution produced is delivered to individual HD stations via a distribution loop.
  CDS is used exclusively in Japan by more than 95% of its HD facilities. It is uncommon outside Japan because of its cost and HD machines outside Japan invariably have built-in proportioning system to handle concentrate instead of dialysate.
  CCS has the lowest cost among the 4 concentrate products. It is predominantly used in the US (by more than 95% of DaVita’s and Fresenius Medical Care’s (FMC) facilities, the 2 largest dialysis providers in US) and Europe (by more than 95% of centres though only for AC).

### Evolution of concentrate production, its cost and risk

In the early days of HD practice in Malaysia, all dialysis facilities produced their own concentrates or dialysates on site. The production was performed manually under open unhygienic condition using crude tools and locally sourced powder salt of poor quality. These backyard produced concentrates were often contaminated and failed to provide consistent solute concentrations. Such backyard productions were abandoned as soon as PPC became widely available in the market in the 1990s. In developed countries however, the early manual unhygienic production has evolved into the modern automated closed production system with ergonomically and purpose designed equipment which minimizes the risk of human error and contamination. The powder salt used to produce the concentrate on-site is of pharmaceutical grade and manufactured under regulated Good Manufacturing Practice (GMP).

There are significant financial and non-financial costs associated with the widespread use of PPC in Malaysia, compare with modern CCS.

- In 2015, the 35,000 patients on HD treatment in the 700+ HD centres in Malaysia [1] are estimated to use about 82 million litres of concentrates at a financial cost of RM130 million including labour and storage space. Payers for dialysis care in Malaysia could save at least RM38 million (29%) a year if all providers were to switch to CCS.
- Production of the 3182 tons of plastic (polyethylene) containers for the PPC and transporting the 89,000 tons of bulky liquid concentrates from the factories to the 700+ HD centres in Malaysia in 2015 has incurred a large energy and carbon footprint. This is estimated to consume as much energy [2] as a sizable Malaysian city of 650,000 people (18,500 Kilowatt-hours per person [3]), and to emit as much CO2 as 130,000 households in Malaysia [4].
- The 3182 tons of plastic containers are ultimately disposed as waste, accounting for about 1% of the 320,000 tons of rigid plastic wastes (3.5% of all wastes) disposed in 2012 in Malaysia [5]. Malaysia is very dependent on landfill to dispose of 95% of its daily 25,000 tons of solid waste, of which only 1 to 5% are recycled [6]. Available space for landfill is becoming scarce and costly, and increasingly met with public protest. Landfill also results in the material and energy stored in plastic being sequestered forever, and the risk of plastic components contaminating ground water is ever present.
- Manual handling of the 89,000 tons of PPC consumed in a year at all provider facilities in Malaysia poses occupational health and safety risk to healthcare workers. Of the 700 extra workers needed in Malaysia to manually handle these loads day-to-day in HD facilities, it is estimated 7 (1%) of them would be afflicted by back injury each year [7].

Besides the above economic costs and externality, microbial contamination of BC is a well-known risk. BC, unlike AC, is supportive of bacterial growth [8], which shows an initial lag phase of up to 72 h when the bacteria adapt to the high salt environment, follow by an exponential growth phase when contamination can reach very high level [9]. In the 1980s, when BC was first introduced to replace acetate buffer, outbreak of pyrogenic reactions were commonly associated with contamination of bicarbonate dialysate [10]. Both PPC and CCS have been implicated. The advent of modern automated CCS however has largely put paid to this concern. In CCS, BC are produced only on demand and consumed the same day over a span of 8 to 12 h, and all unused BC are automatically discarded. In contrast, PPC has a long time lag between production and use which would allow contamination occurring during production, transportation or storage to reach very high level in the BC growth medium. PPC indeed has a long history of FDA mandated product recall since the 1980s, each recall often prompting HD facilities to switch to CCS [11]. The most recent recall [12] was in 2014 which affected Naturalyte, a liquid BC manufactured by FMC at its plant in Montreal Canada to supply its HD facilities in North America. The BC was contaminated by Halomonas (a gram negative bacteria adapted to water with high salt concentration) which resulted in one reported death and 2 injuries, and prompted FDA to designate it the most serious Class 1 recall. FMC has since switched to CCS at almost all its North American HD facilities.

Given such an adverse cost and risk profile, not surprisingly, the use of PPC has long been phased out in developed countries where contamination and occupational safety risk, and environmental cost cannot be ignored or be simply transferred by providers to society and workers. In Malaysia, ironically, the use of PPC, instead of declining in keeping with global trend, has become entrenched with increasingly stringent regulatory proscription of CCS in recent years. This could be due to lack of comparative data [11, 13–21] to support the wider adoption of CCS or more likely providers and policy makers are unaware of the risk and cost of PPC on the one hand, and the safety and performance of modern CCS on the other.

We present here the clinical validation study of the first newly installed modern CCS in Malaysia. While the study was undertaken primarily to investigate its clinical safety and to satisfy local regulatory requirements, we hope that a positive finding will help promote the wider adoption of CCS in Malaysia and other developing countries.

## Methods

We conducted a prospective, randomized, crossover clinical validation study at one HD facility in Malaysia where a modern CCS (HD Integra™, JKS Biomedical) was newly installed to replace an older model in the facility. The study was conducted in accordance with ISO 14155 Clinical Investigation of Medical devices. The Ministry of Health’s Medical Device Authority (MDA) as well as its Medical and Research Ethics Committee (EC) approved the study. The Ethics Committee waived the requirement of informed consent from individual patients since the study units are HD machines rather than individual human subjects. However participating HD centre must inform patients whose HD treatment are to be performed on HD machines identified for the study, and must also in addition obtain their assent by displaying and circulating an information sheet among all patients in the HD centre to inform them that an evaluation of the CCS is ongoing. All approval documents from the MDA and EC are submitted to the journal, while the study protocol and patient information sheet are deposited with the EC.

HD machines included in the study were those used for conventional hemodialysis. These machines used a proportioning ratio of 1 part AC: 1.83 parts BC: 34 parts water. The machines were in good working order and their conductivity measurement calibrated as verified by their maintenance and calibration records. Sixteen HD machines identified by study site as suitable for the study were randomly allocated to a test or reference treatment group (8 per group). Patients on HD treatment with these machines continue to use the same machines throughout the 8-week study. HD machines in the test group used Liquid BC (LBC) which were batch produced on site using the HD Integra™ CCS and distributed through a piping system to individual HD machines for 4 weeks duration. HD machines in the reference group used pre-produced BC (Renasol B, Renal Laboratories Sdn Bhd.) also for 4 weeks duration. Study evaluations occurred twice weekly during the 4 weeks period. Current ISO standards [22–24] require that HD facility monitors CCS weekly until sufficient data have been obtained to demonstrate the adequacy of the system, and thereafter the frequency of monitoring may be reduced to monthly. More intensive monitoring (twice weekly) however is appropriate for the purpose of this study. The primary performance parameters are the measured concentrations of the solutes (sodium, chloride and bicarbonate) in the dialysis fluid produced from the concentrates. They should be present within ±5% of the labelled concentrations (i.e., at nominal concentrations with ±5% tolerance). Samples of the relevant solutes were taken within 1-h into the HD treatment session and the solutes measured according to the recommended test methods described in ANSI/AAMI/ISO. ISO 11663:2009 [23].

Safety assessments consisted of monitoring the concentrates for viable microbial count and measuring their endotoxin level, using the test methods described in ANSI/AAMI/ISO 11663:2009 [23]. Samples of concentrates were obtained in the afternoon of each daily batch production of LBC from the mixing tank, distribution tank and the end distribution loop for this purpose. Data on microbial count and endotoxin level prior to the installation of the upgraded version of HD Integra™ CCS from January 2015 were also retrieved. Occurrence of adverse events in any patients dialyzing on machines used in this study were to be reported by HD facility in accordance with their current Incident Reporting procedure and per current regulatory guideline [25].

After 4 weeks, HD machines originally in the treatment group crossed over to receive the reference treatment, while the reference group similarly crossed over to receive test treatment. Both groups then underwent the test and reference treatments for another 4 weeks duration, during which twice weekly evaluations were also performed. Throughout the 8-week study, neither operators of the HD machines nor other study personnel involved were blinded as it is clearly impossible to mask the 2 treatments.

At the end of the 8-week study, we conducted a survey among users on their experience in using the HD Integra™ CCS. The survey instrument has 55 items to obtain feedback from users on the BC powder packaging, on each step of the operations (water filling, unloading powder, mixing, transfer to holding tank, priming and distribution), end of day shutdown, cleaning and disinfection. Users rate each item on a 5-point scale (1 = poor, 3 = average, 5 = excellent).

### Statistical methods

Sample size was estimated based on the dialysis fluid Sodium concentration as representative solute. Conventional standard [26] for demonstrating bioequivalence of pharmacokinetic parameter requires the 90% Confidence Interval (CI) for the ratio of the mean on test treatment to that on reference treatment to lie within an acceptance range of 0.8–1.25. We used instead a stricter standard for the ratio of the means of Sodium concentration on PPC to CCS to lie within 0.95–1.05, in order to be consistent with the allowed tolerance range of ±5% with respect to the nominal concentration. Assuming the standard deviation (SD) of Sodium is 5.0, for 2X2 crossover study design, beta of 0.1 and alpha of 0.05, the sample size required was 16 HD machines.

The primary performance variable was analyzed for each treatment period (CCS versus PPC) using the mean solute concentration as well as the absolute and relative deviation of the solute concentration from its nominal value. The 95% CI is used to compare the two periods and it should lie within the allowed tolerance range of ±5% of the nominal value.

## Results

Sixteen HD machines were used in the study. Throughout the 8-week study, none of the machines on CCS or PPC arm of the study had alarmed or temporarily ceased functioning as a result of the dialysate conductivity deviating from the preset limit. Likewise no patients reported fever, rigor or other adverse events.

The automated CCS is equipped with variety of sensors and monitors to alert users about system malfunction or potentially hazardous condition, such as when a process is taking longer than expected to complete (time out), or a critical process is not completed properly (eg drainage of RO water or unused concentrates in the system, rinsing and disinfection cycles), or some parameters (such as conductivity, fluid level etc.) have exceeded pre-specified limits. Occurrence of these alerts are automatically logged by the CCS so that the data could be retrieved retrospectively. During the 8-week study, the CCS equipment had sounded an alert on 6 occasions only, and all were due to timeout during the mixing operation (i.e., user has taken longer than the 45 min allowed to fill mixing tank with water and load the powder salt after initiating a batch production).

**Fig. 1:**
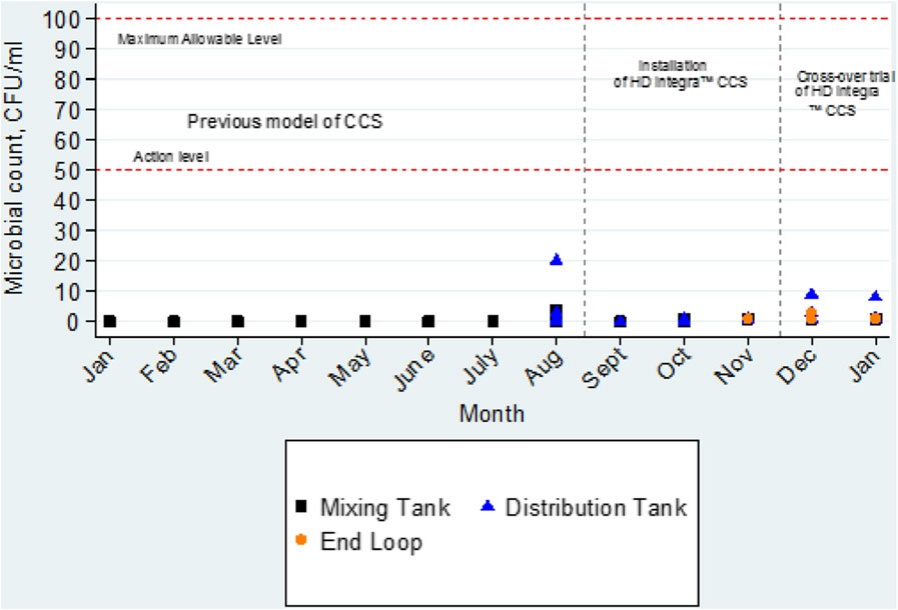
Total viable microbial count in the Mixing tank, Distribution tank and end of Distribution loop at the HD facility between January 2015 and January 2016

**Fig. 2:**
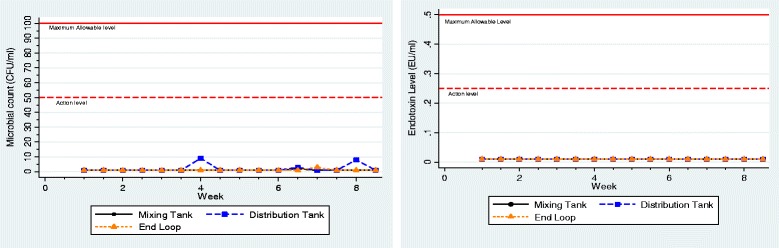
Total viable microbial count and Endotoxin level in the Mixing tank, Distribution tank and end of Distribution loop during the 8-week Clinical validation study

Figure 1 shows the Total viable microbial count in the mixing tank, distribution tank and the end distribution loop prior to installation of the upgraded HD Integra™ CCS when the HD facility was using the previous model (January to August), during installation (August to November) and on 48 occasions during the 8-week cross-over trial (November to January). Throughout all periods, there were occasional non-zero microbial count but they were all below the action level. The endotoxin levels monitored on 48 occasions during the 8-week study were similarly low (Fig. 2).

**Fig. 3:**
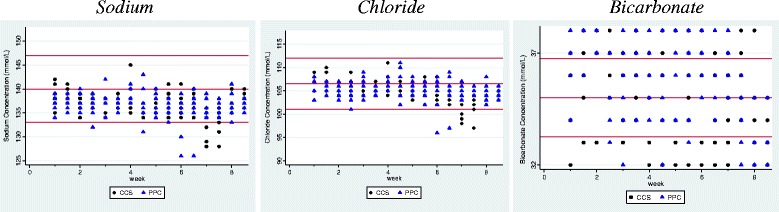
Concentrations of dialysate Sodium, Chloride and Bicarbonate obtained from CCS and PPC over time during the 8-week cross-over study

**Fig. 4:**
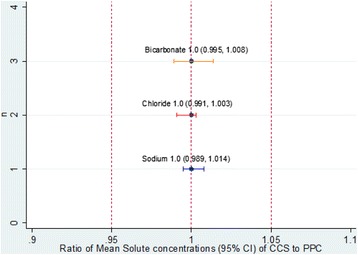
Ratio of Mean Sodium, Chloride and Bicarbonate (95% CI) concentrations of CCS to PPC. *Dotted line* indicates the allowed tolerance limit of ±5%

Dialysate Sodium, Chloride and Bicarbonate concentrations were measured on a total of 128 occasions for each arm of the study. Table 1 compares their mean concentrations on CCS and PPC and Fig. 3 shows their observed values during the 8-week cross-over study. For both CCS and PPC, the mean dialysate Sodium and Chloride concentrations were below the nominal value, the relative deviations range from -1.4 to -2.8% and few observations from either arms exceeded the allowed tolerance limit throughout the study period. The mean dialysate Bicarbonate concentration for both CCS and PPC were close to the nominal value, the relative deviations were 1.4 and 1.2% respectively but the observations are more variable and many more (64 and 71 respectively out of 128 measurements) have exceeded the allowed tolerance limit during the study period. Figure 4 shows the 95% CI for the ratio of the mean Sodium, Chloride and Bicarbonate concentrations on the CCS test treatment to that on the PPC reference treatment. All 3 CIs lie within the allowed tolerance limit of ±5%.

**Table 1:** Comparison of Solute concentrations between Dialysate obtained from CCS and PPC

	CCS	95% CI	PPC	95% CI	Tolerance limits
Study duration, weeks	4	–	4	–	–
Number of HD machines n	16	–	16	–	–
Number of measurements per HD machine	8	–	8	–	–
Total number of measurements	128	–	128	–	–
Sodium					
Nominal concentration N, mmol/L	140	–	140	–	–
E(*Mi* | k) Mean observed concentration ± SD, mmol/L	136.82 ± 2.54	136.13, 137.51	136.62 ± 2.47	135.78, 137.45	133 to 147
E($\overline{\varepsilon i}$ | k) Mean absolute deviation from nominal ± SD, mmol/L	3.38 ± 2.26	2.85, 3.91	3.49 ± 2.31	2.71, 4.27	±7.0
E($\overline{ri}$ | k) Mean relative deviation from nominal ± SD, %	−2.27 ± 1.81	−2.76, −1.78	−2.42 ± 1.76	−3.01, −1.82	± 5%
Maximum absolute deviation from nominal, mmol/L	12	–	14	–	–
Number of observed concentration exceeded allowed tolerance limit	5	–	5	–	–
Chloride					
Nominal concentration N, mmol/L	106.5	–	106.5	–	–
E(*Mi* | k) Mean observed concentration ± SD, mmol/L	104.96 ± 2.23	104.46, 105.46	105.27 ± 2.04	104.72, 105.82	101.17 to 111.82
E($\overline{\varepsilon i}$ | k) Mean absolute deviation from nominal ± SD, mmol/L	2.10 ± 1.69	1.73, 2.47	1.79 ± 1.56	1.38, 2.19	±5.3
E($\overline{ri}$ | k) Mean relative deviation from nominal ± SD, %	−1.43 ± 2.09	−1.90, −0.97	−1.15 ± 1.91	−1.67, −0.63	± 5%
Maximum absolute deviation from nominal, mmol/L	9.5	–	10.5	–	–
Number of observed concentration exceeded allowed tolerance limit	5	–	3	–	–
Bicarbonate					
Nominal concentration N, mmol/L	35	–	35	–	–
E(*Mi* | k) Mean observed concentration ± SD, mmol/L	35.47 ± 1.83	35.23, 35.71	35.41 ± 1.99	35.02, 35.81	33.25 to 36.75
E($\overline{\varepsilon i}$ | k) Mean absolute deviation from nominal ± SD, mmol/L	1.58 ± 1.02	1.30, 1.87	1.77 ± 0.98	1.59, 1.95	±1.7
E($\overline{ri}$ | k) Mean relative deviation from nominal ± SD, %	1.36 ± 5.23	0.68, 2.04	1.18 ± 5.68	0.05, 2.31	± 5%
Maximum absolute deviation from nominal, mmol/L	3	–	3	–	–
Number of observed concentration exceeded allowed tolerance limit	64	–	71	–	–

Seventeen users participated in the user-survey at the end of the study to evaluate the HD Integra™ CCS with respect to its user interface, ease of use, time and labour saving, and reliability. A mean 86% (range 59–94%) of respondents rated these aspects of the CCS performance above average or excellent, and all (100%) rated these average or better. The only concern expressed by users was their rating of the system’s vulnerability to accidental contamination. Ten out of 14 respondents regarded the manual unloading of the powder concentrate into the mixing tank as potentially vulnerable.

## Discussion

There are 2 main challenges to the production and delivery of LBC for HD use. (1) LBC as a bacterial growth medium poses a risk of microbial contamination [8, 9], (2) while the inherent chemical instability of bicarbonate in solution poses a risk of variable solute concentration [27]. This study shows that HD Integra™ CCS, like other modern CCS, was able to overcome both challenges.

### Variation in solute concentration and device performance

This study has shown that the performance of HD Integra™ CCS was statistically equivalent to PPC in terms of measured dialysate solute concentrations. To our knowledge this is the first ever comparative study between concentrates produced using CCS and PPC to demonstrate performance equivalence. The HD Integra™ CCS, like other modern CCS, accomplishes complete dissolution of the powder concentrate by using vortex mixing and high quality salt manufactured under GMP. Gentle vortex mixing and magnetically driven circulation in a closed system also help prevent over-mixing. In regard to the device’s performance in terms of user interface, ease of use, time and labour saving, and reliability, vast majority of users rated the HD Integra™ CCS’s performance above average or better.

### Risk of microbial contamination

This study has also demonstrated the safety of HD Integra™ CCS, in particular its long standing operating record without occurrence of microbial contamination. But clearly contamination did occur from time to time as shown by the non-zero colony count detected (Fig. 1) though the CFU counts have never exceeded the action level. The most important design factors which enables the HD Integra™ CCS to accomplish this level of bacteriological safety are the on demand production of LBC which has to be consumed the same day and the automated discarding of all unused LBC at the end of a treatment day to avoid over-night dwelling of residual LBC. This is in keeping with the observation on microbial growth [9]. Unlike chemical contaminant, it is not enough to achieve low contamination level as bacteria can grow exponentially over time to reach very high level of contamination. Other contributing design features are the daily rinsing cycle and weekly disinfection cycle, which are scheduled automatically. Other modern CCS, but not the HD Integra™, are also equipped with UV light and endotoxin and bacteria retentive filter to produce ultrapure dialysate and substitution fluid for convective therapies [19, 28].

The HD Integra™ CCS as currently designed is certainly not contamination proof. The system is most vulnerable during manual unloading of the powder concentrate into the mixing tank, a vulnerability that is also widely perceived by users. This is the only step in the system operation when its closed system is exposed to the environment and to human, though for less than 5 min, the typical duration taken to unload the powder. The manufacturer did consider automating this step through the use of powder cartridges but this will markedly increase the cost of CCS. However, PPC is also vulnerable when its closed container is opened during HD treatment, and the large number of such opened containers lying on the floor of a HD facility for several hours arguably renders PPC even more risky. This is further aggravated by the high cost of PPC which creates a financial incentive to users to ignore the manufacturers’ instruction to discard leftover LBC (a typical 4-h HD session only consumes about 7.5L of the 10L LBC in a container). Such practices are widespread if not universal in Malaysia, and easily spotted by the salt accretion and corrosion on the floor caused by LBC spillage when leftovers are manually refilled into other containers to make up 10L volume.

### What is to be done?

With 5 domestic manufacturers, PPC will continue to be widely used in Malaysia in the foreseeable future. The least regulator could do is to lift its current exemption from routine microbial surveillance per ISO requirements, as is required for CCS and water treatment system. The presumption that microbial contamination in a HD facility are traceable only to water, CCS and dialyzer reuse, and not to PPC has never been proven. HD providers should also be compelled to follow manufacturer’s instruction to discard all unused LBC. This however would push up cost and will be resisted by providers.

As for CCS, the current proscription on its use should be lifted, which will prompt many manufacturers from US, Japan, Korea and Taiwan to enter this market. However its wider adoption will likely remain slow. Healthcare, as any other businesses, is driven by financial consideration. HD providers will need financially compelling reason to adopt CCS, especially in the Malaysia’s environment where payers are focused solely on keeping reimbursement level low and do not hold providers to account for quality and safety. While the operating cost of CCS is lower than PPC, its adoption will incur significant upfront capital cost in equipment and installation, which will no doubt discourage providers. This study is also limited to a single site and for 8-week duration only. The favourable results will need to be replicated in more sites and for longer duration before one may confidently push for the wider adoption of CCS.

## Conclusion

HD Integra™ CCS, like other modern CCS, is bacteriologically safe and its performance statistically equivalent to PPC. Despite its advantages, its wider adoption will remain challenging.

## Abbreviations

AC: Acid concentrates
BC: Bicarbonate concentrates
CCS: Central concentrate system
CDS: Central dialysate system
CFU: Colony forming unit
CI: Confidence intervals
Cl: Chloride
CO2: Carbon dioxide
FDA: Food and Drug Administration US
FMC: Fresenius medical care
GMP: Good manufacturing practice
HCO3: Bicarbonate
HD: Hemodialysis
LBC: Liquid bicarbonate concentrate
Na: Sodium
PPC: Pre-produced bicarbonate concentrates
